# Histological subtypes and characteristic structures of HPV-associated oropharyngeal carcinoma; study with Japanese cases

**DOI:** 10.1186/1746-1596-8-211

**Published:** 2013-12-19

**Authors:** Mitsuhisa Fujimaki, Yuki Fukumura, Keiko Mitani, Aiko Kurisaki, Junkichi Yokoyama, Katsuhisa Ikeda, Takashi Yao

**Affiliations:** 1 Department of Otorhinolaryngology, Juntendo University, 2-1-1 Hongo, Bunkyo-ku, Tokyo 113-8421, Japan; 2 Department of Human Pathology, Juntendo University School of Medicine, Juntendo University, 2-1-1 Hongo, Bunkyo-ku, Tokyo 113-8421, Japan

**Keywords:** Human papillomavirus-associated oropharyngeal carcinoma, Non-keratinizing squamous cell carcinoma, Abrupt keratinization, Comedo-necrosis among non-maturing island

## Abstract

**Background:**

Human papillomavirus-associated oropharyngeal carcinoma (HPV-OPC) is clinicopathologically distinct entity from the HPV-unassociated one (nHPV-OPC). This study aimed to determine the relationship between histological subtypes of OPC and HPV status for Japanese cases and to identify histological structures of HPV-OPC.

**Methods:**

66 OPC cases were categorized into conventional squamous cell carcinoma (SCC) and the variants. Conventional SCC was subcategorized into keratinizing (KSCC), non-keratinizing (NKSCC), and hybrid SCC (HSCC). HPV status of all cases was determined using p16-immunohistochemistry and HPV-DNA ISH.

**Results:**

Two histological subtypes, NKSCC and HSCC, tended to be HPV-OPC and KSCC tended to be nHPV-OPC with statistical significance. Two histological structures, abrupt keratinization, defined in the text, and comedo-necrosis among non-maturing tumor island, were observed for 58.1% and 38.7% of HPV-OPC, and tended to exist for HPV-OPC with statistical significance.

**Conclusions:**

This study showed the association of NKSCC/HSCC with HPV-OPC in Japanese cases, and two histological structures, abrupt keratinization and comedo-necrosis among non-maturing island, were considered characteristic histological features of HPV-OPC.

**Virtual slides:**

The virtual slide(s) for this article can be found here: http://www.diagnosticpathology.diagnomx.eu/vs/1816432541113073.

## Background

Human papillomavirus (HPV)-associated oropharyngeal carcinoma (HPV-OPC) has been recognized as a distinct clinicopathologic entity of head and neck cancer [1]. HPV-OPC has been reported to be distinct from HPV-unassociated oropharyngeal carcinoma (nHPV-OPC) on a molecular, epidemiological, and clinical basis [2,3]. From a clinical perspective, HPV-OPC is likely to be more radio-/chemo-sensitive, with a longer recurrence-free period, more positivity in regional lymph nodes, and with better prognosis than nHPV-OPC [4-7]. As for the pathological view, p53 overexpression/mutation are found at significantly lower frequency for HPV-OPC than for nHPV-OPC [1,8].

In terms of histopathology, there have been several papers on some histological subtypes/variants that are more likely to be HPV-associated [9-15]. For example, Chernock et al. reported that non-keratinizing SCC (NKSCC) and hybrid SCC (HSCC) were more likely to be HPV-positive than keratinizing SCC (KSCC) [9,10]. However, there has been no study using Japanese cases, or no study focusing on characteristic histopathological structures for HPV-OPC so far. In Japanese OPC cases, the impact of several risk factors other than HPV infection, such as tobacco/alcohol exposure, may be different from that in Western countries. For example, in Western countries, patients with HPV-OPC are reported to have less tobacco exposure but more marijuana exposure than those with nHPV-OPC [3,5]. It is important to see the tendency of histological subtypes of HPV-OPC in Japanese cases with the different background risk factors.

In this study, we classified our HPV-OPC and nHPV-OPC cases by histological subtypes and determined if there are some histological features of concern, in order to determine the specificity/sensitivity of NKSCC, KSCC, HSCC for HPV-OPC with Japanese OPC cases and to identify histological structures characteristic for HPV-OPC.

In this study, HPV status for each case was judged by p16 immunohistochemistry (IHC)/in situ hybridization (ISH). Although there have been studies which propose genomic amplification of human telomerase gene or C-MYC as helpful screening methods for HPV-associated cancer/high-grade lesions in cases of uterine cervix [16,17], a gold standard method for detecting HPV-associated OPC has not been established at present. Several studies have mentioned the reliability and accessibility of p16 IHC and ISH for HPV DNA [18-21]. In this study, we used the combined detection method of HPV ISH and p16 IHC according to Singhi et al. [19].

## Methods

### Cases

**Figure 1:**
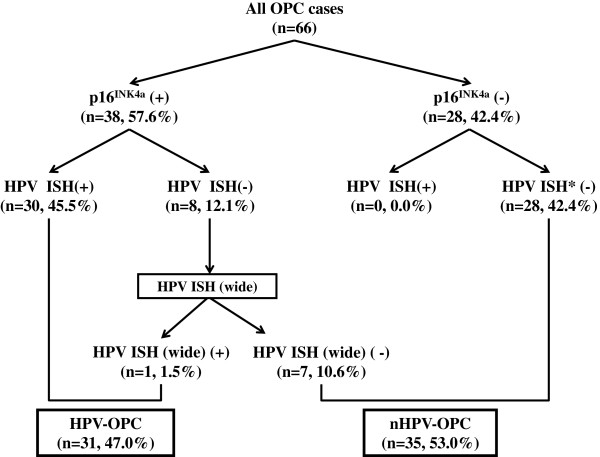
**An algorithm for the judgment of high-risk HPV association with oropharyngeal carcinoma.** Cases positive for both p16 IHC and ISH were judged as high-risk HPV-associated oropharyngeal carcinoma (HPV-OPC). ISH specific for type 16/18 HPV was conducted for all cases. Cases discordantly p16 positive by IHC but HPV16/18 negative by ISH were further evaluated for wide-spectrum HPV types (types 16, 18, 31, 33, 35, 39, 45, 51, 52, 56, 58, 59, and 68). Number of cases corresponding to each is shown in parentheses.

**Figure 2:**
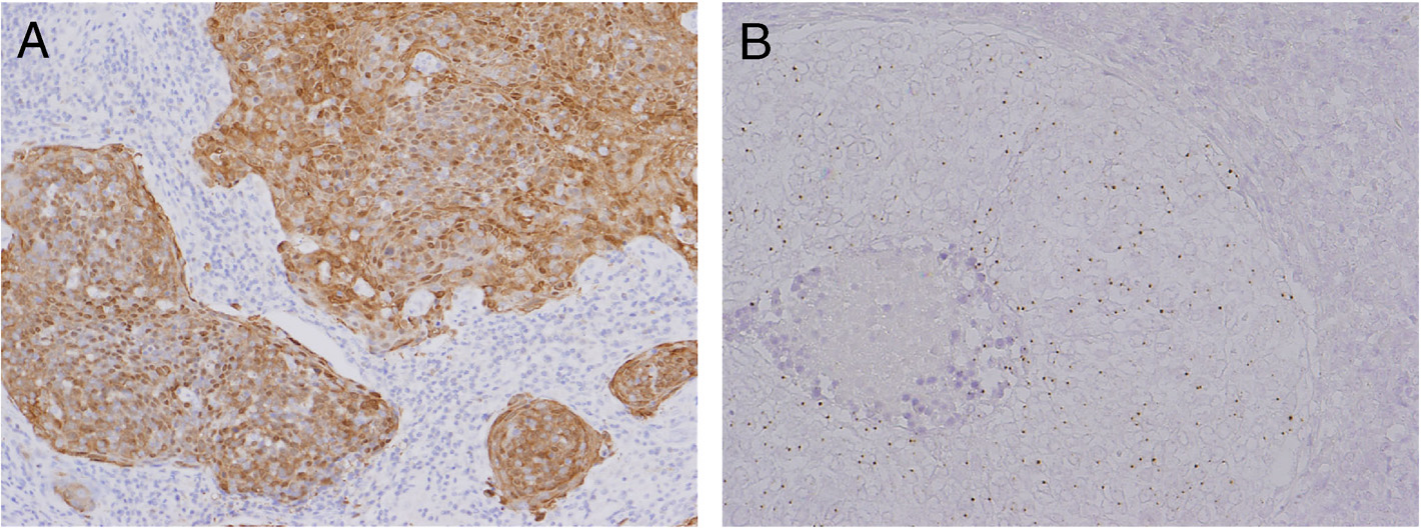
**Judgment for p16 immunohistochemistry (IHC) and In situ hybridization (ISH) for high- risk HPV-DNA. (A)** A representative p16 IHC-positive case, showing intense nuclear and cytoplasmic staining for more than 70% of tumor cells. **(B)** A representative ISH positive case for high-risk HPV-DNA, showing hybridization signals visualized as punctate dots. [**(A)** IHC for p16, original magnification, x200, **(B)** ISH for HPV-DNA, original magnification, x300].

66 pathological specimens of previously untreated oropharyngeal carcinoma (OPC), of which 39 were biopsy and 27 were surgical specimens, were studied. All patients were treated in the Department of Otorhinolaryngology, Head and Neck Surgery, Juntendo University, between 2004 and 2012. All materials were obtained before chemo- or radiotherapy. The clinical data, including tobacco/alcohol exposure, tumor site, clinical stage, and HPV status of cases, are shown in Table 1. The HPV status was judged according to a previous study [19]. Briefly, we used the result of IHC for p16 and that of ISH for HPV-DNA as shown in our algorithm (Figure 1). For IHC of p16, the result was interpreted as positive when more than 70% of tumor cells showed intense levels of both nuclear and cytoplasmic/nuclear staining (Figure 2A). For ISH, cases were interpreted as positive if hybridization signals visualized as punctate dots were present in the nuclei of the squamous cells (Figure 2B). The methods of ISH and IHC for p16 are described below.

**Table 1:** Clinical features of oropharyngeal SCCs

	**Total**	**%**	**HPV-OPC**	**%**	**nHPV-OPC**	**%**	**P value**
	**(n = 66)**		**(n = 31)**		**(n = 35)**		
Sex							
Male	44	66.7	21	47.7	23	52.3	NS
Female	22	33.3	10	45.5	12	54.5	
Age^a^	66(37–90)		66.5( 43–87)		67.0(37–90)		NS
Tobacco^b^							
(-)	29	43.9	13	44.8	16	55.2	NS
(+)	37	56.1	18	48.6	19	51.4	
Alcohol^b^							
(-)	29	43.9	12	41.4	14	58.6	NS
(+)	37	56.1	19	51.4	21	48.6	
Site^c^							
Lat	50	75.6	27	54.0	23	46.0	NS
Ant	11	16.7	4	36.4	7	63.6	
Post	3	4.5	0	0.0	3	100.0	
Sup	2	3.2	0	0.0	2	100.0	
Stage							
I/II	11	16.7	6	54.5	5	45.5	NS
III/IV	55	83.3	25	45.5	30	54.5	

^a^Age: mean and range in parenthesis are shown

^b^Tobacco and alcohol: (-), never; (+), ever and current

^c^Tumor site: Lat, lateral wall; Ant, anterior wall; Post, posterior wall; Sup, superior wall.

### Assessment of histopathology of oropharyngeal SCC

**Figure 3:**
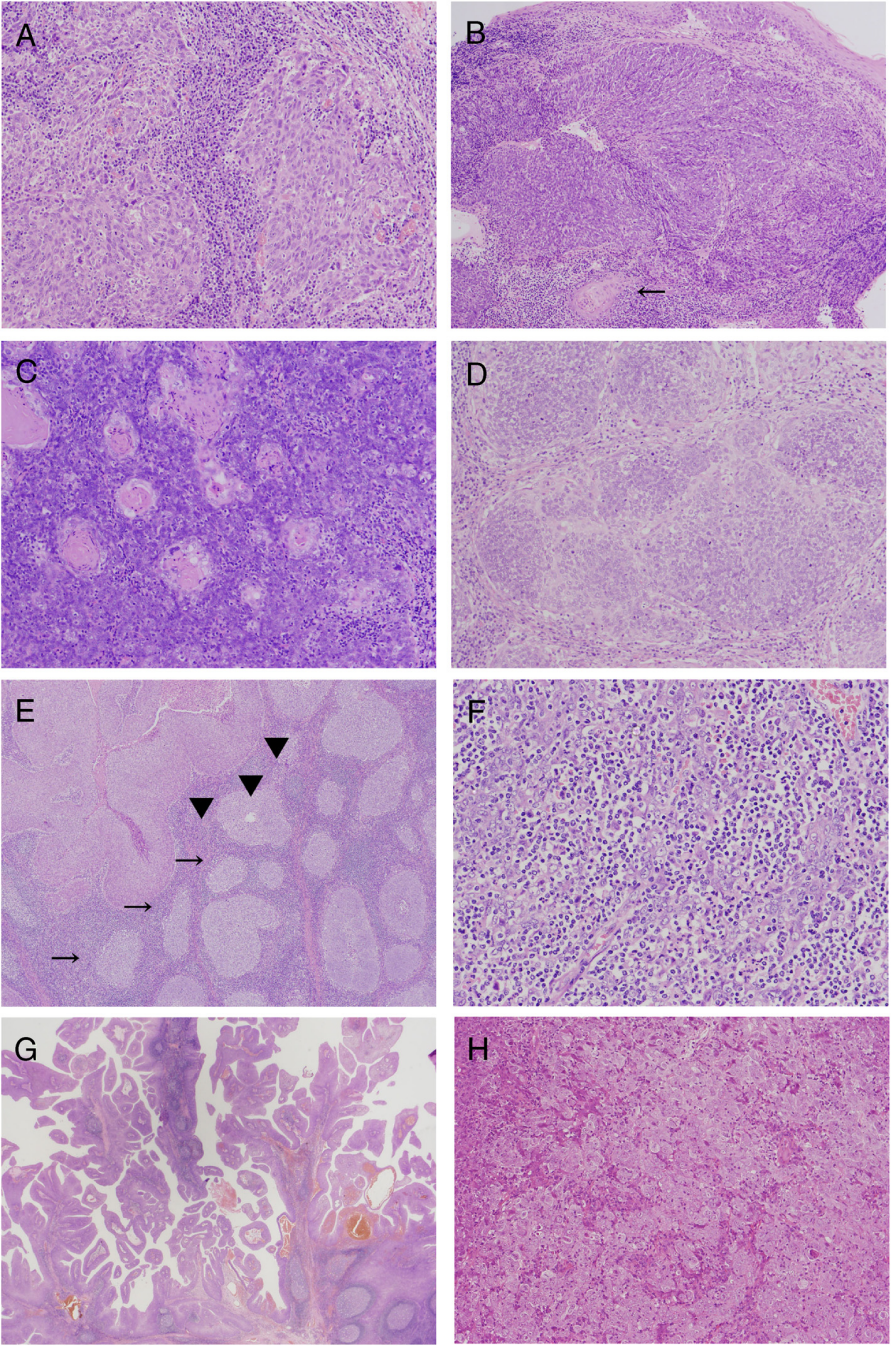
**Histological classification. (A)** Representative cases of keratinizing squamous cell carcinoma (KSCC): The tumor was composed entirely of mature squamous cells. **(B)** Representative cases of nonkeratinizing squamous cell carcinoma (NKSCC): Tumor cells form sheets of nonmaturing cells with pushing borders, only sometimes with peripherally or abruptly keratinizing/maturing focus (Arrow). **(C & D)** Representative cases of hybrid squamous cell carcinoma (HSCC): Tumor grows in non-maturing cell sheet, but with focal squamous maturation diffusely throughout the tumor. **(E)** Representative case of basaloid squamous cell carcinoma: Small and densely packed tumor cells and with grow in a lobular configuration. Basaloid SCC (arrows) was seen next to conventional NKSCC (arrow heads). **(F)** Representative case of lymphoepithelial carcinoma: Tumor cells have syncytial cytoplasm, growing with prominent inflammatory background. **(G)** Representative case of papillary squamous cell carcinoma: Tumor cells grow in an arborizing papillary fashion. **(H)** Unclassified squamous cell carcinoma: The tumor cells, showing bizarre nuclei with high nuclear grade, grow monotonously; hence, this could not be classified into any of the above. [**A** to **H**, Hematoxylin and eosin staining, with original magnification: x200, x150, x200, x200, x150, x300, x100, x300 , respectively].

The histopathological classification and assessment of the tumors were conducted using light microscopy by three of the authors (MF, YF, and AK), in a blinded manner and without knowledge of HPV status. Formalin-fixed, paraffin-embedded samples were cut into 4-um sections and stained with hematoxylin and eosin (H&E). One to 5 sections for each were reviewed. For the histological classification, we followed the definitions in the WHO classification/previous articles [9-15,22], briefly, (i) KSCC, defined as being entirely composed of mature squamous cells without areas of NKSCC morphology, as described below (Figure 3A), (ii) NKSCC, defined as forming sheets with pushing borders of hyperchromatic, non-maturing tumor cells (Figure 3B), (iii) HSCC, showing NKSCC morphology and containing areas of focal squamous maturation throughout the tumor (Figure 3C,D), (iv) basaloid SCC (Figure 3E), (v) lymphoepithelial carcinoma (Figure 3F), and (vi) papillary SCC (Figure 3G). When cases could not be classified into any of the above, these were categorized as unclassified SCC (Figure 3H). When there was a mixture of two histological types, such as NKSCC and basaloid SCC, the case was categorized as the variant in this study (hence, in this case, as basaloid SCC).

**Figure 4:**
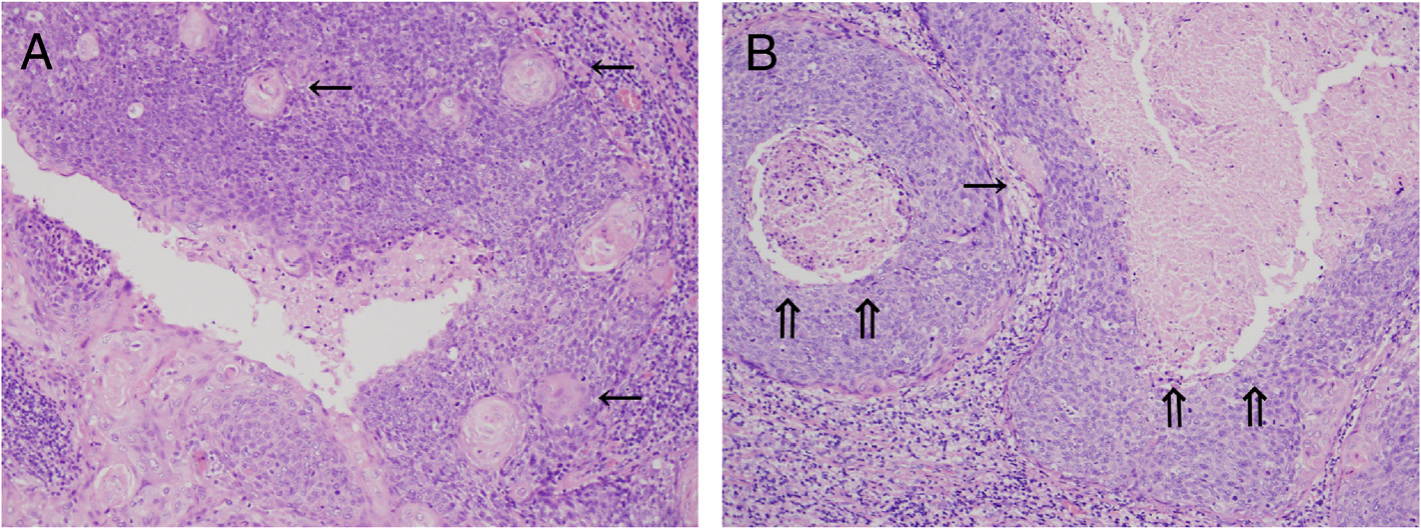
**Abrupt keratinization and Comedo-necrosis among non-maturing tumor nest. (A)** A representative HPV-OPC case with abrupt keratinization (arrows); some are located peripheral to tumor nest and others are among non-maturing tumor cells. **(B)** A representative HPV-OPC case with comedo necrosis among non-maturing tumor nest (double arrows); coagulative necrosisis seen among non-maturing tumor cell nest. A single arrow indicate abrupt keratinization. [**A & B**, Hematoxylin and eosin staining with original magnification, original magnification, x200 for both].

Since two histopathological features, (i) ‘abrupt keratinization’ and (ii) ‘comedo-necrosis among non-maturing tumor island’, were often seen in our OPC cases, but were usually not observed in our daily pathological practice for squamous cell carcinoma of any other organ/tissue, we carefully evaluated the existence of these two histological structures. Here, we defined (i) ‘abrupt keratinization’ as cancer pearl formation or evident squamous maturation among or peripheral to the non-maturing tumor cell nest (Figure 4A) and (ii) ‘comedo-necrosis among non-maturing tumor island’ as coagulative necrosis formed among non-keratinizing/non-maturing tumor cell nest (Figure 4B). The existence of each histological parameter of ‘abrupt keratinization’ or ‘comedo-necrosis among non-maturing tumor island’ was analyzed independently.

### Immunohistochemistry (IHC) for p16

IHC for p16 was performed using 4-μm-thick tissue sections from formalin-fixed paraffin-embedded tissues and with DAKO EnVision™ + Kit (DAKO Corp., Carpenteria, CA) following the manufacturer’s instructions.

#### In situ hybridization (ISH) for high-risk HPV-DNA

For ISH, a catalyzed signal amplification method for biotinylated probes (DAKO GenPoint, Carpenteria, CA) was used. Briefly, 5-μm tissue sections underwent deparaffinization, heat-induced target retrieval in citrate buffer (DAKO Target Retrieval Solution, Carpenteria, CA), and digestion using Proteinase K (DAKO, Carpenteria, CA). Slides were subsequently hybridized with a biotinylated HPV16/18-type-specific probe (DAKO, Carpenteria, CA). Signal amplification was performed by consecutive application of streptavidin-HRP complex and biotinyl tyramide. Visualization of positive hybridization signals was performed by incubation with the chromogenic substrate diaminobenzidine. Interpretation of staining was performed without knowledge of p16 IHC positivity or tumor origin. In some cases where there was discordance between p16 IHC and HPV 16/18 status (i.e. in p16 IHC-positive, ISH-negative cases), we performed analysis with a more extended panel of ISH probes that included types 16, 18, 31, 33, 35, 39, 45, 51, 52, 56, 58, 59, and 68 (DAKO, Carpenteria, CA) in order to check for other high-risk HPV associations.

As controls, squamous cell carcinoma of the tonsil was hybridized with a positive control (Human DNA, DAKO, Carpenteria, CA) biotinylated DNA probe, and with a negative control (Plasmid DNA, DAKO, Carpenteria, CA) biotinylated DNA probe.

### Statistics

To determine the association of each of the histological subtypes/histological structures with HPV status, and to see the relationship of patients’ age (43-59/60-87) and sex (F/M) to histological subtypes of OPC, cross-tabulation analyses were performed using Fisher’s exact test. The statistical analyses were performed using PASW Statistics 18.0 (SPSS, Chicago, IL).

### Ethical Approval

This study was approved by the ethics committee of Juntendo University School of Medicine.

## Results

### Histopathological features of HPV-OPC

Among all the 66 OPC cases examined, 54 (81.8%) were categorized as conventional SCC, being subcategorized into 22 (33.3%), 21 (31.8%), and 11 (16.7%) cases of KSCC, NKSCC and HSCC [Table 2]. Both NKSCC and HSCC cases were much more likely to be HPV-OPC than KSCC (Fisher’s exact test, both with p < 0.01), with sensitivity/specificity of 65.2%/80.6% for NKSCC and 30.4%/87.1% for HSCC. On the other hand, the sensitivity/specificity for KSCC being predictive of nHPV was 67.7%/95.6%. There were no association statistically between patients’ age/sex and histological subtypes of HPV-OPC. The cases other than conventional SCC were basaloid, papillary, and lymphoepithelial SCC, and were found in 3 (4.5%), 2 (4.5%), and 2 (3.0%) OPC cases, respectively, and the former two variants were more frequently HPV-OPC [Table 2].

**Table 2:** Histological classification of oropharyngeal carcinomas

**Histological type**^**a**^	**Number of cases**	**HPV-OPC **^**b**^	**nHPV-OPC**^**c**^	***P *** **value**
Conventional SCC				
KSCC	22(33.3%)	1(4.5%)	21(95.5%)	
NKSCC	21(31.8%)	15(71.4%)	6(28.6%)	<0.01
HSCC	11(16.7%)	7(63.6%)	4(36.4%)	<0.01
Variant				
Basaloid SCC	3(4.5%)	3(100.0%)	0(0.0%)	
Papillary SCC	3(4.5%)	2(66.7%)	1(33.3%)	
Lymphoepithelial carcinoma	2(3.0%)	1(50.0%)	1(50.0%)	
Unclassified	4(6.1%)	2(50.0%)	2(50.0%)	
Total	66	31(47.0%)	38(57.0%)	

^a^Histological Type: KSCC, keratinizing SCC; NKSCC, nonkeratinizing SCC; HSCC, hybrid SCC.

^b^HPV-OPC, HPV-associated oropharyngeal carcinoma.

^c^nHPV-OPC, HPV-unassociated oropharyngeal carcinoma.

Abrupt keratinization, as defined above, was observed in 18/31 HPV-OPC cases (58.1%) and in 2/35 nHPV-OPC cases (5.7%). Comedo-necrosis among non-maturing tumor island was observed in 12/31 nHPV-OPC cases (38.7%) and in 1/35 of nHPV-OPC cases (2.9%). The existence of abrupt keratinization/comedo-necrosis among non-maturing tumor island was both statistically associated with HPV-OPC (Fisher’s exact test, both with p < 0.001) [Tables 3 and 4].

**Table 3:** Abrupt keratinization in oropharyngeal carcinomas

**Histological type**^**a**^	**Number of cases**	**HPV-OPC **^**b**^	**nHPV-OPC**^**c**^	***P *** **value**
Conventional SCC				
KSCC	0/22(0%)	0/1(0%)	0/21(0%)	
NKSCC	13/21(61.9%)	11/15(73.3%)	2/6(33.3%)	
HSCC	7/11(63.6%)	7/7(100.0%)	0/4(0%)	
Variant SCC	0/12(0%)	0/8(0%)	0/4(0%)	
Total SCC	20/66(30.3%)	18/31(58.1%)	2/35(5.7%)	<0.001

**Table 4:** Comedo-necrosis among non-maturing tumor island in oropharyngeal carcinomas

**Histological type**^**a**^	**Number of cases**	**HPV-OPC **^**b**^	**nHPV-OPC**^**c**^	***P *** **value**
Conventional SCC				
KSCC	0/22(0%)	0/1(0%)	0/21(0%)	
NKSCC	8/21(38.1%)	6/15(40.0%)	2/6(33.3%)	
HSCC	4/11(36.4%)	4/7(57.1%)	0/4(0%)	
Variant SCC	0/12(0%)	1/8(12.5%)	0/4(0%)	
Total SCC	14/66(21.2%)	12/31(38.7%)	2/35(5.7%)	<0.001

## Discussion

The present study showed that most (87% of all cases and 90% of biopsy cases) OPC cases were conventional SCC, and were subcategorized into 36.7% KSCC, 31.7% NKSCC, and 18.3% HSCC, where NKSCC and HSCC were more likely to be HPV-OPC, and most KSCC was nHPV-OPC with statistical significance. These results for Japanese OPC cases are in accordance with those of Chernock et al. [9]. In the study by Chernock et al., they compared the frequency of each of the three subtypes and the result of ISH for HPV-DNA and p16 IHC, and their result of ISH seemed to be more similar to our present data than that of p16 IHC.

In the present Japanese study, no significant difference in clinical data, including risk factors, was seen between HPV-OPC and nHPV-OPC groups. In Western countries, patients with HPV-OPC have different epidemiological data including less tobacco exposure, more marijuana exposure, and lower age than those with nHPV-OPC [3,5]. It seems interesting that, despite the different clinical background, there are still similar tendencies of histological subtypes between Japanese and Western HPV-OPC.

A few researchers have reported the association of papillary, basaloid, and undifferentiated SCC (lymphoepithelial SCC) with HPV-OPC [11-15]. In this study, there was also a tendency of the former two variants to be HPV-OPC, although with a small sample size.

When investigating the histopathological features of HPV-OPC, some histological structures were noteworthy. Abrupt keratinization, cancer pearl formation/squamous maturation in an abrupt fashion among non-mature tumor island, or at a peripheral site of tumor nest, were not usually observed in our daily pathological practice for squamous cell carcinoma of any other organ/tissue. Although comedo-necrosis is not rare in many carcinomas including keratinizing SCC, comedo-necrosis among non-maturing SCC cells is unusual. Hence, we analyzed whether these two structures are characteristic histological structures for HPV-OPC or not. This study suggested that these two histological structures, abrupt keratinization and comedo-necrosis among non-maturing tumor island, are very specific for HPV-OPC.

## Conclusions

Our study showed that NKSCC and HSCC are statistically more likely to be HPV-associated carcinoma, while KSCC is statistically more likely to be nHPV-OPC, which was also true for Japanese patients. Two histological structures, abrupt keratinization and comedo-necrosis among non-maturing island, were considered to be very specific pathological structures for HPV-OPC.

### Consent

Written informed consent was obtained from the patient for the publication of this report and any accompanying images.

## Competing interests

The authors declare that they have no competing interests.

## Authors’ contributions

JY and KI conceived the study idea and designed the study. MF, YF, and AK reviewed the literature, performed in situ hybridization and statistical analyses. KM performed immunohistochemistry. MF and YF extracted data and drafted the manuscript. TY reviewed and edited the manuscript. All authors read and approved the final manuscript.
